# Microplastics in Airborne Particulate Matter: A Comprehensive Review of Separation Techniques, In Vitro Toxicity and Health Impacts

**DOI:** 10.3390/ijms262110332

**Published:** 2025-10-23

**Authors:** Dominika Uchmanowicz, Katarzyna Styszko, Xijuan Chen, Giulia Terribile, Rakshit Jakhar, Giulio Sancini, Justyna Pyssa

**Affiliations:** 1 Faculty of Energy and Fuels, AGH University of Kraków, Al. Mickiewicza 30, 30-059 Kraków, Poland; duchmanowicz@agh.edu.pl (D.U.); jakhar@agh.edu.pl (R.J.); jpyssa@agh.edu.pl (J.P.); 2 Sino-Spain Joint Laboratory for Agricultural Environment Emerging Contaminants of Zhejiang Province, College of Environmental and Resource Sciences, Zhejiang Agriculture and Forestry University, Hangzhou 311300, China; chenxj@zafu.edu.cn; 3 Human Physiology Unit, School of Medicine and Surgery, University of Milano-Bicocca, 20900 Monza, Italy; giulia.terribile@unimib.it (G.T.); giulio.sancini@unimib.it (G.S.)

**Keywords:** MPs, toxicity, air pollution, particulate matter, health impact

## Abstract

Microplastics (MPs) are emerging airborne pollutants that can migrate through various environmental pathways, with air representing one of the most critical exposure routes. Their occurrence within suspended particulate matter (PM)—a major atmospheric pollutant associated with respiratory, cardiovascular, and neurological diseases—further amplifies the risks posed by air pollution. The main sources of airborne MPs include tire and road wear, degradation of larger plastic debris, and wind-driven resuspension from soil and landfills. This review provides a comprehensive synthesis of current knowledge on airborne MPs, integrating methodological and toxicological perspectives. It summarizes sampling and separation procedures (filtration, chemical digestion, density separation) and analytical techniques for qualitative and quantitative identification. Particular emphasis is placed on the toxicological implications of MPs, including oxidative stress, inflammatory responses, and potential carcinogenicity, as revealed by in vitro and mechanistic studies. In light of the absence of standardized methodologies, this work highlights the urgent need for harmonized protocols linking environmental monitoring with biological toxicity assessment. By combining information on analytical workflows and cellular responses, this review serves as a key reference for developing environmentally relevant experimental designs and evaluating health risks associated with airborne microplastics. It therefore bridges the gap between environmental analysis and toxicological research, outlining future priorities for methodological standardization and risk assessment.

## 1. Search Strategy and Study Selection

The literature search was conducted between March and October 2025 using the databases Scopus, Web of Science, and ScienceDirect. The following keywords and Boolean combinations were applied: “microplastics” OR “airborne microplastics” OR “atmospheric microplastics” OR “tire wear particles” OR “brake wear particles” OR “PM-bound microplastics” AND “toxicity” OR “in vitro” OR “cell culture” OR “exposure”.

The search was limited to peer-reviewed articles and reviews published mostly between 2020 and 2025. Additional references were identified from the bibliographies of relevant papers.

Inclusion criteria covered studies addressing (i) the occurrence and sources of airborne MPs, (ii) analytical and separation methods for MPs in air and particulate matter, and (iii) toxicological effects assessed through in vitro or in vivo approaches. Exclusion criteria included conference abstracts, non-peer-reviewed reports, and studies focused solely on aquatic or soil environments without atmospheric relevance.

## 2. Introduction

### 2.1. Background and Significance

Despite numerous scientific reports and studies on plastics, particularly MPs, the science of synthetic polymers remains one of the most revolutionary research fields. As an inexpensive, long-lasting and versatile material, plastic is used worldwide across nearly every industrial sector and human activity [1]. Its unique properties including low thermal conductivity, a high strength-to-weight ratio and durability contribute to an estimated annual production and consumption of approximately 300 million tons [2,3,4]. As larger plastic objects and materials fragment, particles with a diameter of less than 5 mm are formed, commonly referred to as MPs. These particles can appear as fibers, fragments, or films [3]. They have been detected in soils, sewage sludge, surface and groundwater, air, and living organisms [5].

Microplastic pollution poses significant health risks to both humans and the environment, with the problem intensifying due to the strong adsorptive properties of MPs particles. These particles exhibit a high specific surface area and pronounced hydrophobicity [6]. Although their affinity for adsorbing organic pollutants is lower than that of activated carbon, plastic particle surfaces can still adsorb various harmful substances, including polycyclic aromatic hydrocarbons (PAHs), pharmaceuticals, endocrine-disrupting compounds, hydroxylated derivatives, and other toxic pollutants [7,8,9,10,11]. This further exacerbates the environmental impact of microplastic contamination [3]. MPs, which are inherently harmful, can interact with organic and inorganic pollutants present in the environment. Depending on factors such as particle type, size, aging state, temperature, pH, salinity, and contact time, the risk of adsorbing additional micropollutants may vary [3,12,13]. These interactions further amplify the issue, escalating it into a large-scale environmental concern [3].

### 2.2. Primary and Secondary MPs

Based on their origin, MPs can be classified as primary (commercial) or secondary MPs. Primary MPs are deliberately produced for direct or indirect application as precursor materials in the manufacture of various consumer polymer products. They are commonly found in cosmetic and personal care items such as facial washes, exfoliating scrubs, toothpastes, and abrasive formulations, as well as in industrial processes, including washing of synthetic textiles, rubber manufacturing, and even tea bag production. Moreover, primary MPs are used in the medical field as carriers for active substances in drug delivery systems [14]. Currently, about 45 types of plastics are produced and utilized worldwide, with the most common being polypropylene (PP), polyethylene (PE), polyethylene terephthalate (PET), polystyrene (PS), polyurethane (PU), polyvinyl chloride (PVC), and polycarbonate (PC) [14].

In the natural environment, primary MPs undergo various transformations, turning into secondary MPs (which represent the most abundant type found in the environment) with modified surface structure and adsorptive properties [14]. Key factors influencing these changes include ultraviolet (UV) radiation, water-induced corrosion, and temperature fluctuations [3]. The sources of secondary MPs are diverse and span multiple industrial sectors.

### 2.3. Environmental Pathways and Ecological Impacts

Due to the high mobility of MPs, these particles can spread uncontrollably across the environment over long distances, particularly through atmospheric transport by wind, as well as via industrial and domestic wastewater [15]. Small fibers from textiles, particularly sportswear, can be shed and released directly or indirectly into the environment during wear or washing [16]. MPs particles found in cosmetics are small enough to pass through urban sewage systems during rinsing, thus bypassing the initial filtration stages in wastewater treatment plants.

Once released to aquatic systems, MPs become a major ecological concern because ingestion by marine biota causes direct, physical injury. Documented effects include mechanical abrasion and ulceration of the gastrointestinal epithelium; gut blockage and impaction leading to reduced feeding, false satiation, and negative energy balance; decreased filtration efficiency and valve/gill dysfunction in bivalves; mucus hypersecretion and lamellar damage in fish gills with impaired gas exchange; and impaired buoyancy and swimming performance due to altered gut contents and increased drag. At the base of the food web, MPs can enter algal cells, where they inhibit growth and photosynthesis. These physical injuries propagate through trophic transfer, amplifying impacts along the food chain [2,6,17]. In animals, such injuries have been linked to intestinal damage, body-condition loss (weight loss), liver lesions/necrosis, and-at high burdens-mortality [3].

### 2.4. Tire Wear Particles (TWPs) and Traffic-Related Emissions

**Figure 1:**
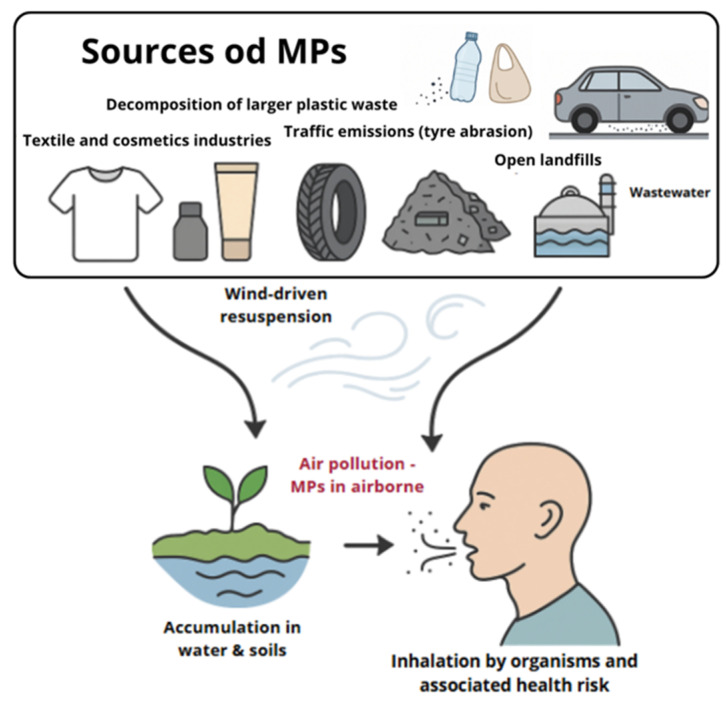
Sources, pathways and atmospheric transport of MPs.

Air, as a crucial vector for pollutant transport, carries MPs particles both in outdoor and indoor environments [16,18,19]. MPs are particularly prevalent in suspended particulate matter, including PM10, PM2.5, and PM1 [20]. The circulation of MPs in the environment and their incorporation into suspended particulate matter are illustrated in Figure 1.

One of the primary sources of these pollutants in the environment is transportation, where MPs particles have been proven to originate from tire wear on road surfaces [15,21]. The latest research by Özen and Mutuk (2025) has revealed the presence of MPs in PM10 particulate matter samples collected near highly trafficated urban kerbsite [20]. The most common kind was fragments (51.43%), followed by filaments (37.14%) and fibers (11.43%) [20]. This indicates that vehicle traffic, particularly tire wear, contributed significantly to the problem [20]. A substantial proportion of the black MPs particles were cigar-shaped and cylindrical, like tire abrasion particles. Particles ranged from 159.98 to 1090.76 μm, with the most common size class being 0.25–0.5 mm (42.86%), indicating the breakdown of larger polymers [20]. The most common and significant color (42.86%) was black, most likely because of tire wear. MPs from tires, largely consisting of polybutadiene, were produced because of road friction, brake wear, and abrasion [20].

Car tires are composed of a combination of natural and synthetic rubbers, which transform into MPs when subjected to mechanical wear. Modern tires are mostly composed of petroleum-based synthetic rubbers, with additional ingredients like zinc oxide (1%) working as a catalyst and sulfur (1–4%) for vulcanization. Furthermore, carbon black (22–40%) is frequently used as a filler to improve UV resistance. Additionally, oils that are typically aromatic are applied to enhance wet grip and flexibility. These substances disperse into tiny particles, which pollute the environment. The behavior of these particles in water is influenced by the specific gravity of tire rubber, which determines whether they sink or remain afloat [22]. Özen and Mutuk (2025) identified five different types of polymers in air samples collected from the university, according to FT-IR analysis: polyethylene (PE) (73%), polypropylene (PP) (14%), polybutadiene (PB) (9%), polystyrene (PS) (4%), and polyamide (PA) (5%) [20]. PE and PP were commonly found in a variety of items, including automobile parts. Notably, a significant portion consisted of polybutadiene (PB), a key component of car tires, indicating significant release from tire wear, brake wear, and road friction [20]. PS, commonly used in packaging and insulation, and PA, found in textiles and automotive parts, were also present. Carbon and oxygen were found in the Energy-Dispersive X-ray Spectroscopy (EDS) analysis, as well as trace metals such as barium, zinc, aluminum, and iron, which are commonly employed as plastic additives [20]. The interaction between tires and road surfaces generates tire wear particles (TWPs), a major source of MPs pollution [12,22].

Airborne MPs from car tires are mostly composed of tire wear particles (TWPs), which are produced as the outcome of friction between tires and road surfaces during vehicle movement [21]. The abrasion process produces MPs pieces made of synthetic rubber, natural rubber, and other chemical additives, such as styrene–butadiene rubber (SBR) and butadiene rubber (BR) [12,23]. These particles combine with road dust and can be resuspended in the air because of vehicle-induced turbulence, wind, and weather [12]. Furthermore, heat buildup during tire–road contact causes some compounds to volatilize, resulting in ultrafine MPs particles entering the environment [24]. These particles are released into the air, rainwater runoff, and road dust, spreading across various environmental compartments. Some contribute to inhalable PM10 pollution, which is widespread and highly transportable over long distances by air and water [12].

The distribution of TWP is further influenced by seasonal variations, such as the increased bitumen wear particles in winter brought on by the usage of studded tires [12]. Traffic-derived MPs, which can be resuspended and carried by wind or water, are abundant in road dust. Even at lower concentrations in airborne samples, TWP still pose the risk of environmental deposition and long-distance transport [25]. The amount of MPs emitted from tires is influenced by a variety of factors, including tread compound, size, vehicle characteristics (e.g., mass, wheel alignment), road characteristics (e.g., surface type), driving style (e.g., acceleration, braking), and environmental conditions (e.g., temperature) [12,26]. Heavy vehicles, as well as those with more lateral and longitudinal acceleration, wear their tires faster. Road surface roughness and temperature fluctuations also influence emission rates, with higher ambient temperatures frequently resulting in less wear [26].

According to studies, front-wheel-drive automobiles have more front-tire abrasion, whereas rear-wheel-drive vehicles have more rear-tire wear. Furthermore, because of their increased weight, electric vehicles have greater abrasion rates than internal combustion vehicles [26]. Although data on heavy-duty vehicles is limited, they are expected to emit substantially more MPs per vehicle than passenger automobiles [26]. Tire wear particles (TWPs) can disperse and accumulate in various environmental conditions [12]. TWPs can remain suspended in the air for extended periods after being released, depending on particle size and other environmental conditions [12,26]. Larger particles typically settle on roadways, soils, and water bodies through dry or wet deposition, whereas smaller particles transport further before settling. Rain and surface runoff also contribute to the long-term accumulation of TWPs in aquatic environments by transferring them into rivers, drainage systems, and oceans [12]. The transport dynamics of these particles change over time due to weathering, fragmentation, and interactions with organic materials. Due to their chemical composition, TWPs are highly persistent, resisting degradation and accumulating in both urban and rural environments [23].

TWPs, which include MPs, contain hazardous compounds including zinc, phthalates, and antioxidants [14]. The chemicals released from these particles into the environment affect aquatic life, including fish, crabs, and algae, leading to oxidative stress, energy depletion, and developmental issues [12]. Because MPs have a large surface area, they readily absorb pollutants, making them more hazardous, especially at the nanoscale. Furthermore, TWPs alter soil properties and release toxic leachates that harm plants, animals, and soil microbes [12]. To evaluate the health impacts of TWPs, Jiand and colleagues created a 3D in vitro human airway organoid (hAO) model [27]. Exposure to TWPs lead to reduce hAO size and to increase cell apoptosis, oxidative stress, inflammation, and an imbalance in airway cell types such as basal, ciliated, goblet, and club cells. These findings suggest that TWPs may contribute to lung illness by compromising airway function [27].

### 2.5. Exposure Pathways and Potential Health Relevance

As suspended PM enters organisms, MPs migrate alongside it. Studies indicate that PM particles smaller than 5 µm can readily penetrate the human body, with PM1 being of particular concern. This fraction can reach vital organs such as the lungs, potentially causing immediate and harmful health consequences [28]. Notably, suspended particulate matter, along with MPs, can settle on floor surfaces. Frequent hand-to-mouth contact particularly among crawling infants heightens the risk of ingestion and subsequent absorption into their bodies [16]. Further research on the occurrence of MPs remains essential for expanding our understanding of their ongoing environmental consequences associated with their presence [29]. Issues related to MPs, their migration pathways in the natural environment, and their ability to adsorb various micropollutants on their surfaces are therefore of critical importance [1,30]. The risk of inhaling MP particles due to water, soil, and air contamination warrants special attention, given the scale of global plastic production [16].

### 2.6. Monitoring Approaches and Research Gaps

In addition to traditional techniques for detecting MPs in the air, which are discussed in detail in this study, it is worth noting at the outset that natural biomonitors can also be used to assess the presence and concentration of airborne pollutants, including MPs [31]. Biomonitors such as leaf surfaces can trap MPs from the atmosphere, reducing their concentration in the air. The efficiency of MPs filtration varies depending on the tree species, leaf type, and other factors [31]. Studies have shown that leaves can accumulate MP fibers, fragments, and granules, with concentrations ranging from 0.07 to 0.19 particles/cm^2^. Another example is mosses, where research has found MP fibers ranging in size from 30 to 5000 µm, with smaller particles below 400 µm being dominant [31]. Similarly, lichens, particularly those found in polluted areas such as landfills, can accumulate MP debris, mainly in the range of 7 to 79 particles per gram. Active air samplers also include honeybees, which collect MP particles during foraging flights [31]. Fibers and fragments of MPs have been found on their bodies. Additionally, spider webs can capture airborne MPs. Analysis of spider webs has revealed the presence of various types of plastics, including polyethylene terephthalate (PET), polyvinyl chloride (PVC), tire wear particles, and polyethylene [31].

The use of biomonitors includes assessing the atmospheric dispersion of pollutants, monitoring the presence of MPs in the air, and identifying pollution sources [31]. Biomonitoring can serve as an alternative to traditional air and sediment sampling methods.

Due to the lack of standardized analytical methods for the identification and separation of MPs from environmental samples, there is a continuous need to systematize knowledge and expertise in this field. This study primarily focuses on discussing separation methods for MPs from suspended particulate matter collected from the air, aiming to support further research on their harmful effects. MPs analysis requires a multi-step approach, including sampling, separation, characterization, and quantification [5].

The present review provides a comprehensive synthesis of current knowledge on the occurrence, characterization, and toxicological impact of airborne MPs, with particular emphasis on their human health implications. This work integrates methodological aspects of sampling, separation, and identification with mechanistic toxicology and in vitro exposure studies. In light of the current lack of methodological standardization, this review highlights the need for unified approaches linking environmental monitoring with cellular-level toxicity assessment.

This paper serves as a key knowledge compendium that bridges the gap between environmental and biological research perspectives, supporting the development of harmonized experimental designs and realistic exposure models for future risk assessment of airborne MPs.

## 3. Methods for Microplastic Separation from Suspended Particulate Matter (PM10)

### 3.1. Air Sampling

To investigate the presence of MPs in suspended particulate matter, proper sample collection is essential. Various approaches are employed for this purpose, including high-volume active air samplers, which use vacuum pressure to draw air through a chamber, capturing particles on filters. This method allows for flexible airflow control and ensures high precision in trapping both particles and vapors [32]. Passive samplers are also used to collect MPs from the surrounding air over time, relying on natural air currents. These samplers operate without active pumping systems, making them cost-effective and suitable for long-term environmental monitoring. By passively accumulating airborne MPs, they provide valuable data on the distribution and deposition rates of these pollutants in different environments. Passive samplers are also employed, collecting MPs from ambient air over time by relying on natural air currents. However, their effectiveness may be lower in areas with very low or high pollutant concentrations, potentially limiting their accuracy [32]. Additionally, road and household dust can be collected using brushes, vacuum cleaners, or active sampling methods, though these are generally less precise than atmospheric deposition samplers [33]. Another innovative approach involves the use of drones and unmanned aerial vehicles (UAVs) equipped with specialized measurement instruments. These devices can collect samples at various altitudes, providing valuable insights into the vertical distribution of MPs [5].

For flow-based samplers, filter selection is tailored to retain the desired particle size, such as PM10 or PM2.5. It is crucial that the filters are made from materials free of synthetic polymers to prevent contamination and ensure accurate analysis. Various filter types exist, though fiberglass and polycarbonate filters are not recommended for MPs studies due to their potential to releasing contaminants. During the sampling stage, meticulous documentation is essential, including details such as the sampler’s location, filter diameter, pore size, and any pre-treatment steps taken before placing the filter in the sampler. The pre-treatment process is designed to minimize or eliminate potential contaminants by heating the filters at high temperatures or rinsing them with appropriate solvents.

Contaminants that may be present on filter surfaces and must be removed include various ambient particles that could compromise the reliability of subsequent analyses. Proper storage of filters before and after sampling is also crucial. To prevent contamination, plastic containers should be avoided, as they may introduce unwanted substances onto the filter surfaces [28].

### 3.2. Separation of MPs from Suspended Particulate Matter Samples

The separation of MPs particles from air samples primarily involves filtration, serving as the fundamental step for extracting solid particles from the air. Following filtration, the collected samples undergo preparation for further analysis. However, it is important to note that separation processes for atmospheric samples present unique challenges due to the extremely fine nature of the collected material and the high risk of contamination at every stage of analysis. Contaminants can originate from various sources, including measurement equipment, laboratory materials, and even ambient air. To minimize these risks, strict procedural controls must be implemented, including maintaining contamination-free laboratory environments, employing proper sample handling techniques, and conducting blank sample analyses. These measures help distinguish genuine MPs from potential external contaminants, ensuring the reliability and accuracy of the results [5].

Studies on the MPs content in suspended particulate matter begin with the separation of MPs from the environmental matrix, which involves the removal of organic contaminants and interfering substances prior to further analysis. This is achieved through methods such as filtration, organic matter digestion, density separation, and electrostatic techniques [5,28].

The digestion process can involve alkalis, acids, enzymes, or chemical reagents such as hydrogen peroxide (H_2_O_2_), potassium hydroxide (KOH), Fenton’s reagent (H_2_O_2_ + Fe(II)), and sodium hydroxide (NaOH) are commonly used. These chemicals effectively remove organic matter, with hydrogen peroxide being the least harmful to MPs [34]. Acids such as nitric acid (HNO_3_), hydrochloric acid (HCl), perchloric acid (HClO_4_), and sulfuric acid (H_2_SO_4_) can also be employed; however, they pose a risk of MPs degradation. Enzymatic digestion, using cellulase, lipase, and protease, offers a milder approach that does not alter MPs [34]. The choice of reagent is crucial, particularly for subsequent Raman spectroscopy analysis, as certain chemicals may affect the integrity of MP particles and influence spectroscopic results.

After digestion, MPs are isolated using density separation, a technique in which MPs float to the surface in a solution of appropriate density, while heavier components settle at the bottom [34]. Commonly used solutions for this method include sodium chloride (NaCl), calcium chloride (CaCl_2_), sodium iodide (NaI), and zinc chloride (ZnCl_2_) [34,35]. Sodium chloride, with a density ranging from 1.15 to 1.30 g/cm^3^, is a cost-effective and widely used option, though it is ineffective for high-density polymers such as PET or PVC. Calcium chloride is often chosen due to its efficiency and availability [35]. If a high proportion of dense polymers is suspected in suspended particulate matter, sodium iodide or zinc chloride may be used, despite their higher costs and potential environmental impact.

To enhance efficiency, a density column is preferred over a traditional separatory funnel, as it minimizes clogging and facilitates the extraction of MPs from the upper section of the column. Additionally, fluorescent dyes are employed to improve visualization and monitoring of the extraction process, ensuring precise separation and identification of MPs [35]. Electrostatic techniques rely on the differences in electrical properties between MPs and dust particles. This method is based on electrostatic charging of particles and the application of electrostatic forces to separate them. MPs can be modified with magnetic particles, allowing them to adhere to surfaces due to their electrostatic charge, while dust particles remain unchanged and separate upon the application of magnets [5]. The main advantage of this method is that it avoids the use of aggressive chemicals, preserving the physical and chemical properties of MPs. It is more environmentally friendly and less destructive to the surfaces of separated particles. The efficiency of this method depends on ambient humidity and the specific material properties of the MPs. Potential limitations may arise based on the type of analyzed matrix, but this method is not yet fully understood [5].

In summary, the mentioned digestion and separation methods for MPs vary in their effectiveness in removing sample matrix while minimizing polymer degradation [28]. During separation, material loss may occur, especially with small particles, and the materials used in the process may introduce contamination into the samples [36].

To facilitate comparison between protocols, Table 1 summarizes the main approaches used for sampling, digestion, and density separation of microplastics, including the reagents employed, their performance, and associated QA/QC challenges.

### 3.3. Quantitative and Qualitative Analysis of MPs

Characterization of MPs includes the identification of their shape, size, color and chemical composition. Visual microscopic methods, often in combination with staining, are used to identify MPs in PM samples collected from the air. Binocular, fluorescence and Scanning Electron Microscopy coupled with Energy-Dispersive X-ray Spectroscopy (SEM-EDS) are used for this purpose. For polymer identification, infrared spectroscopy (FTIR), Raman spectroscopy and thermogravimetry combined with mass spectrometry (TGA-MS) are used [33].

Another category of methods to identify polymers and their concentrations is the use of thermochemical techniques. One of these is gas chromatography–mass spectrometry (GC/MS) pyrolysis. The combination of pyrolysis, gas chromatography and mass spectrometry enable comprehensive analysis of MPs, enabling the identification of polymer (e.g., PET, PVC, PE) and the determination of their concentration in the sample. It is worth noting, however, that this method is destructive, i.e., the sample is destroyed during the process, preventing further analysis of the same sample by other techniques. This method also does not allow the number of particles or their shape to be determined. Thermochemical techniques also include the GC/MS coupled thermal desorption method. This is a more advanced technique that allows the analysis of samples with very small amounts of MPs, combining thermal desorption with GC/MS [33].

Both FTIR and Raman spectroscopy are based on the interaction of electromagnetic radiation with molecular bonds and are widely used for polymer identification and mapping of microplastic particles. The practical detection limit of conventional FTIR techniques, including ATR-FTIR, is approximately 50 µm, while µFTIR systems equipped with focal plane array (FPA) or mercury cadmium telluride (MCT) detectors can theoretically detect particles as small as 10 µm [26]. In practice, however, the reliable detection limit for environmental samples is closer to 20 µm, due to diffraction limitations and signal attenuation caused by surface weathering and irregular morphology, leading to an underestimation of particles below this threshold [26]. In contrast, µRaman spectroscopy offers higher spatial resolution, with a theoretical diffraction limit of approximately 250 nm and practical detection capabilities down to 1–2 µm, depending on the laser wavelength and fluorescence intensity.

However, Raman analysis may be affected by fluorescence interference and particle heating, especially for colored or aged polymers. The use of near-infrared lasers (785 nm) can mitigate fluorescence but reduces scattering intensity, which may affect the detection of smaller MPs [33]. Additionally, the use of appropriate filters is essential to avoid interference from the substrate. Aluminum oxide (Anodisc) filters with pore size of 0.2 µm are most frequently used, as polymer-based filters (PTFE, polycarbonate) can produce background absorption bands that obscure spectral signals [33].

Spectral identification criteria also play a crucial role in the accuracy of polymer classification. Most studies recommend using a Hit Quality Index (HQI) or correlation coefficient (r) of 0.70–0.80 as the minimum threshold for reliable polymer identification. Lower matching scores can lead to misclassification, particularly for aged, oxidized, or biofilm-covered particles. Manual verification of spectra near the threshold and cross-validation with complementary spectroscopic techniques (e.g., µFTIR and µRaman) are therefore essential for robust QA/QC [33].

Results are normalized to MPs concentrations in air (particles/m^3^) and dust (particles/g or mg/g). Methods for the analysis of MPs vary in terms of complexity and accuracy. It is important to use as many analytical techniques as possible to gain information on both the number of MPs particles, their mass and their morphology and polymer composition [5,28]. All the above-mentioned methods for separation and analysis of MPs from airborne particulate matter are not standardized, which poses a problem for the comparability of results from different studies [34]. The need for standardization is strongly signaled by academics, but the topic is further along in development [5,28,33].

## 4. Human Health Impact of MPs in PM

Suspended particulate matter in the atmosphere has been characterized as one of the main air pollutants, posing a significant threat to human health. Numerous epidemiological studies indicate that exposure to suspended particulate matter is associated with an increase in the number of disease cases, primarily respiratory and cardiovascular diseases. Several studies have investigated the cellular and molecular effects of MPs on respiratory and epithelial models. Table 2 summarizes the key experimental findings, reported endpoints, and major limitations of previous work on MPs-induced toxicity, emphasizing gaps in environmental relevance and methodological consistency.

Short-term exposure to suspended particulate matter may lead to a sudden deterioration in well-being, while long-term exposure contributes to the development of chronic obstructive pulmonary disease (COPD) and increases the risk of lung and laryngeal cancers. Moreover, the risk of acute myocardial infarction, cardiac arrhythmias, heart failure, and bronchial asthma increases, particularly among the elderly [47,48].

Suspended particulate matter is classified based on fractions: PM10, which includes particles smaller than 10 µm; PM2.5, consisting of particles smaller than 2.5 µm; and the most dangerous fraction, PM1, which contains particles smaller than 1 µm. The smaller the particles that make up atmospheric aerosol, the greater the threat, due to the ease with which fine particles penetrate directly into the respiratory system of living organisms. PM2.5, due to the small size of its particles, penetrates deep into the respiratory system, causing inflammation and damaging tissues, which increases the risk of serious diseases [47,48]. An increase in the concentration of suspended particulate matter in the air (particularly PM2.5 and PM1) is associated with a rise in hospitalizations, heart rhythm disturbances, heart attacks, and, in some cases, can even lead to death [6,49].

Suspended particulate matter is a variable and complex mixture of solid particles and gases, originating not only from typical combustion processes but also from other sources of air pollution [49]. Suspended particulate matter consists of various substances, such as sulfates, nitrates, organic matter, elemental carbon, heavy metals like lead and cadmium, as well as trace elements (Ca, Al, Na, Cl, P, S, and O). Studies have shown that suspended particulate matter serves as a carrier of various pollutants, with MPs potentially being one of them. Exposure to suspended particulate matter is therefore associated with simultaneous exposure to MPs and other harmful substances, further exacerbating the issue of air pollution [47]. This means that MPs can be inhaled along with the air, entering the respiratory system [6,28,49].

Sources of MPs in the air include the breakdown of larger plastic waste, wind lifting MPs from soil and landfills, tire wear (up to 84% of airborne plastic comes from road dust), textile fibers released from drying synthetic fabrics and everyday clothing use, emissions from industrial processes, burning in household stoves, and beauty salons, where MPs are emitted during manicure procedures [6,28,48]. MPs particles in the atmosphere vary in size and chemical composition, which may affect their toxicity [6,50]. Among the identified MPs are polyethylene terephthalate (PET) and polyethylene (PE) [28]. The movement of MPs in the air is determined by transport, dispersion, and deposition processes, which are proportional to their size, shape, and length. Smaller particles, including nanoparticles, are more susceptible to long-range transport than larger fragments. Particles in the form of fibers and fragments have different aerodynamic properties compared to spherical particles [51]. MP particles are primarily transported by wind, which lifts them over various distances, and by air currents, which enable their dispersion across different atmospheric layers [28,51]. To identify the characteristics of MPs that contribute to their potential harmfulness, their physicochemical properties and interactions with the environment are considered. However, it is important to emphasize that the processes of particle transport in the atmosphere are dynamic and complex, and MPs can move between different environmental components, such as air, water, and soil, making it difficult to control and accurately assess their impact and presence [28,48,51].

Human exposure to MPs occurs predominantly through inhalation, ingestion and, to a lesser extent, dermal contact. Once introduced into the human body, MPs can translocate to various organs and tissues, such as lungs, the gastrointestinal tract, placenta, the bloodstream and even the brain [52].

According to Prüst and colleagues (2020), exposure to micro- and nanoplastics induces neurotoxic effects including oxidative stress, acetylcholinesterase inhibition, neurotransmitter imbalances, and behavioral alterations across various models. They emphasize the urgent need for systematic studies comparing the effects of different particle types, sizes, and exposure conditions to better assess associated neurotoxic risks [53].

**Figure 2:**
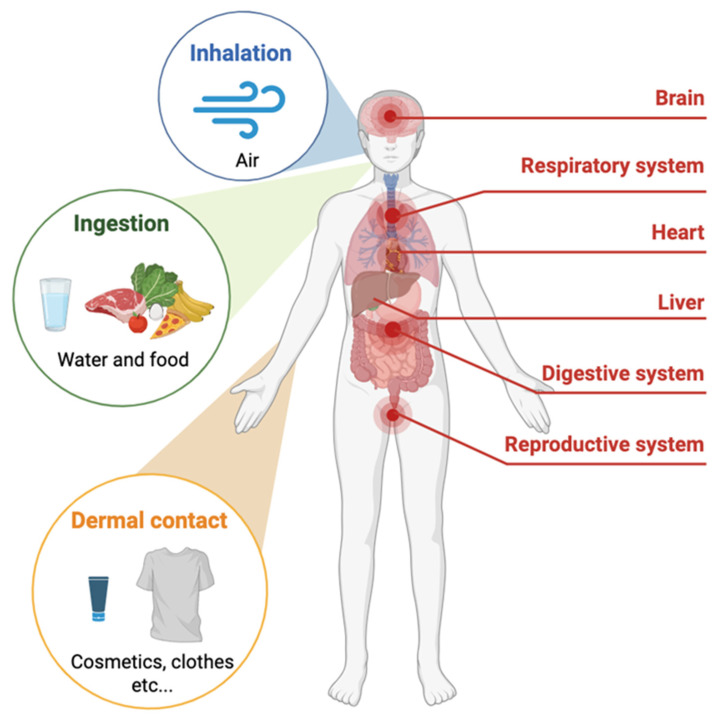
Human health impact of MPs in PM.

The potential impact on human health is of increasing concern. MPs have been shown to elicit pro-inflammatory responses, oxidative stress, immune dysregulation, and disturbances in the gut microbiota. These biological effects may contribute to a range of health outcomes, including gastrointestinal dysfunction, respiratory irritation, metabolic disorders, endocrine disruption, reproductive toxicity and neurological dysfunctions (Figure 2).

Furthermore, due to their physicochemical properties-such as high surface area and hydrophobicity-MPs can act as carriers for toxic environmental contaminants (e.g., heavy metals, polycyclic aromatic hydrocarbons and persistent organic pollutants), potentially enhancing their bioavailability and toxicity [53]. Exposure to MPs, especially through inhalation, can lead to their accumulation in various organs of the body, potentially resulting in negative health consequences. MPs act as a harmful stimulus to the body, causing cellular disturbances, inflammation, metabolic abnormalities, and an increased risk of cancer [6].

In vitro studies show that MPs particles induce oxidative stress and inflammatory responses in lung epithelial cells, as well as disrupt lung barrier function [6]. They can also lead to changes in gene expression, triggering cell death (apoptosis) and causing an inflammatory response. Long-term exposure to MPs may reduce the lungs’ ability to repair damage. Once MPs enter the respiratory tract, they activate inflammatory cells, damaging tissues and increasing the risk of chronic obstructive pulmonary disease (COPD).

Recent studies have highlighted the potential for inhaled MPs to deposit within human lung airways, where they may induce inflammation, oxidative stress, and compromise pulmonary function. Despite growing concern, the mechanistic understanding of MPs interactions with respiratory tissues remains limited. This underscores the urgent need for further research to elucidate the cellular and molecular pathways underlying MPs-induced lung toxicity and to assess the long-term health implications of chronic exposure [54,55]. Additionally, MPs can interact with other pollutants, such as phthalate esters (PAEs), which may enhance their toxicity [6]. Animal studies indicate that MPs can also cause pulmonary fibrosis by activating oxidative stress and the Wnt/β-catenin signaling pathway. They have been found to disrupt reproductive cells in the ovaries and cause damage to the eye surface [13,56]. MPs such as polystyrene (PS) and polyethylene (PE) can reduce cell viability and alter inflammatory and oxidative stress markers in in vitro studies on MDCK (canine kidney epithelial) cells and L929 (mouse fibroblast) cells [56].

Although most evidence to date derives from in vitro studies and animal models, the implications for human health are profound, particularly in vulnerable populations such as pregnant women and children [52,57]. Epidemiological data are currently limited, and long-term effects remain largely uncharacterized [14]. Consequently, comprehensive studies are urgently needed to assess chronic exposure, determine safe threshold levels, and understand the mechanisms underlying MPs-induced toxicity in humans [14,51,53].

### 4.1. In Vitro Studies on the Carcinogenicity of MPs

**Figure 3:**
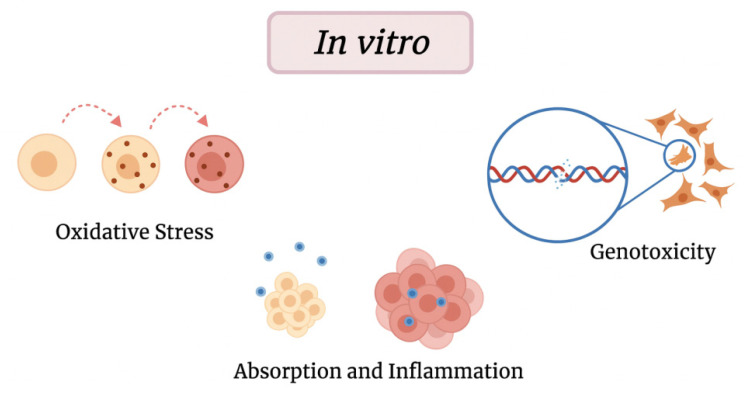
In vitro effects of MPs.

In vitro studies (on cells) allow for a direct assessment of the impact of MPs on cells shown in Figure 3 in terms of oxidative stress, changes in gene expression, inflammatory markers, and DNA damage [13,58].

Various types of cells can be used for this purpose, such as lung epithelial lines (A549, BEAS-2B), are commonly used for this purpose [13,39,50]. Oxidative stress is a well-established factor contributing to cancer development, as MPs can generate reactive oxygen species (ROS) leading to lipid peroxidation, protein oxidation, and DNA strand breaks. Both direct and indirect DNA damage may result from MPs exposure, potentially causing mutations and promoting carcinogenic processes [13,14,58]. In vitro studies can reveal how MPs affect the expression of genes associated with cancer-related processes, including the induction of apoptosis (cell death), inflammatory responses, and signaling pathways [58]. Mechanistic toxicology studies have revealed that the toxicity of MPs and nanoplastics (NPs) is closely related to the activation of specific signaling pathways, including Nrf2, NF-κB, and MAPK cascades [14]. Exposure to PS-NPs and TWPs has been consistently shown to induce excessive production of reactive oxygen species (ROS), triggering oxidative stress and downstream molecular responses [12,39]. Excessive ROS generation suppresses the Nrf2–Keap1 antioxidant defense system, limiting the transcription of key detoxifying enzymes such as SOD1/2, CAT, GPx1, and HO-1, while simultaneously activating NF-κB—a central pro-inflammatory transcription factor responsible for upregulating IL-1β, IL-6, IL-8, and TNF-α expression [14,39,50]. This redox imbalance disrupts mitochondrial integrity and promotes cytokine release, leading to chronic inflammation and tissue injury. Concurrently, MPs induce the activation of the MAPK (ERK, JNK, and p38) pathway, which modulates oxidative stress responses and apoptosis [14,39,50]. MAPK-dependent phosphorylation of transcription factors (e.g., AP-1, HIF-1α) enhances inflammatory gene transcription and contributes to mitochondrial dysfunction and caspase-mediated apoptosis. Upregulation of genes encoding antioxidant enzymes (SOD2, HO-1) and pro-apoptotic mediators (BAX, caspase-8/9, DR5, cytochrome c) further indicates that oxidative stress and apoptosis are tightly coupled cellular outcomes of MPs/NPs exposure [39,50]. In addition, ferroptotic responses have been proposed as an emerging mechanism of MP-induced cytotoxicity, driven by iron-dependent lipid peroxidation and depletion of intracellular glutathione [42]. The activation of ferroptosis-related genes, often accompanied by suppression of the Nrf2–GPX4 axis, links oxidative imbalance to irreversible membrane damage and necrotic cell death [58].

Recent advances have demonstrated that these interconnected mechanisms-oxidative stress, inflammation, MAPK activation, apoptosis, and ferroptosis-can be triggered even at sub-toxic or environmentally relevant concentrations [14,41]. At such low exposure levels, MPs and NPs have been shown to alter the expression of antioxidant and inflammatory genes without inducing overt cytotoxicity, suggesting an early disruption of cellular homeostasis [14,40]. In vitro experiments using realistic exposure doses (e.g., 1 ng/cm^2^ of PS-NPs) in BEAS-2B bronchial epithelial cells have revealed upregulation of NF-κB, NLRP3, and ROCK-1, indicating that environmentally relevant concentrations can already activate pro-inflammatory and oxidative pathways [41].

Moreover, to better mimic real-world respiratory exposure scenarios, advanced in vitro models such as the air–liquid interface (ALI) and human airway organoids (hAOs) have become essential for modeling realistic respiratory exposure to airborne MPs and NPs. ALI cultures of A549 and Calu-3 cells allow for monitoring transepithelial electrical resistance (TEER) and tight-junction proteins (e.g., ZO-1, occludin), serving as sensitive indicators of early epithelial barrier impairment [12,13,27,38,40,41]. Even in non-cytotoxic conditions, PS-NPs and TWPs were reported to induce compensatory upregulation of ZO-2 and pro-inflammatory cytokines (IL-6, IL-8, TNF-α), reflecting adaptive but stressed barrier responses [12,14,40,41,54]. Similarly, exposure of hAOs to tire wear particles disrupted basal (KRT5) and club (SCGB1A1) cell marker expression, confirming impaired differentiation and tissue remodeling at doses near environmental relevance [27].

These findings underscore that mechanistic pathways activated by MPs and NPs-including Nrf2/NF-κB/MAPK signaling, apoptosis, and ferroptosis-are not exclusive to acute exposures but can also occur under chronic, low-dose, environmentally relevant conditions [27,41,44]. Such insights highlight the necessity of integrating advanced in vitro systems (ALI and organoids) and mechanistic biomarkers (ROS generation, TEER, cytokine profiling) to evaluate realistic human health risks associated with airborne MPs [12,14,39]. Some of the key findings on MPs toxicity, including polymer type, particle size, applied dose units, exposure models, biological endpoints, and study limitations, are summarized in Table 3. The comparison highlights that toxic responses depend strongly on physicochemical characteristics (size, surface charge, and functionalization), exposure conditions, and the applied biological model. While submerged cultures typically reveal general cytotoxic and oxidative responses, more advanced systems-such as Transwell, ALI, and organoid models-provide mechanistic insights into epithelial barrier impairment, inflammatory signaling, and apoptosis at environmentally relevant concentrations [27,39,41,44,45,50,58,59]. The summarized studies also emphasize the lack of methodological standardization, variability in dose metrics (µg·mL^−1^, ng·cm^−2^, mg·L^−1^), and the need for chronic, low-dose exposure assessments to better approximate real-world inhalation scenarios.

### 4.2. Preparation of Material for In Vitro Studies

In analyzing the impact of air pollution on health, particularly through in vitro studies, proper preparation of the extract is essential to reflect real exposure conditions [47]. MPs samples collected from suspended particulate matter should undergo appropriate separation processes, as discussed in the previous chapter. After conducting qualitative and quantitative analyses using appropriate methods (also described in the previous chapter), the preparation of the suspension for in vitro studies can begin. The preparation of the suspension involves dispersing solid particles in a suitable cell culture medium, such as saline solution, phosphate-buffered saline (PBS), or other specialized media. To prevent particle agglomeration, ultrasonication is applied, followed by sterilization to eliminate potential contamination of the cell culture [50].

For in vitro studies, human lung epithelial cell lines, such as A549, BEAS-2B, HPAEpiC, and HNEpCs, as well as airway organoids, are most used [13,50]. Cells are exposed to the suspension under controlled conditions, with exposure doses and duration adjusted to the study’s objectives [50]. Various exposure methods are employed, such as submerging the cells in suspension or more advanced techniques like ALI exposure, which better simulates respiratory exposure conditions [33]. After exposure, parameters such as cell viability, oxidative stress, cytokine production, and gene expression are analyzed [50].

In vitro studies are closely linked to epidemiological research, so the particle concentrations used in in vitro experiments should correspond to exposure levels observed in the population [47,48]. The results are analyzed in the context of epidemiological studies to understand the mechanisms of harmful factors at the cellular and molecular levels, providing deeper insight into the health effects of air pollution [47,48]. Epidemiological studies, utilizing time-series analysis and regression models, examine the relationship between pollutant concentrations and the incidence of various diseases, as well as the increase in relative risk [33,47,48].

### 4.3. Potential Errors Affecting Toxicological Results

Interpretation of toxicological data on MPs, NPs, and TWPs is often complicated by methodological inconsistencies and non-standardized experimental conditions. Several key factors can significantly influence observed toxic responses in vitro and in vivo [14].

The physicochemical properties of tested particles-such as size, shape, surface charge, and degree of aging-strongly determine their cellular interactions [54]. Most studies rely on spherical, pristine PS-NPs, which do not reflect the heterogeneous, weathered particles found in the environment [54]. Aging processes (e.g., UV exposure, oxidation) alter surface chemistry and hydrophobicity, often enhancing ROS production and inflammatory signaling compared with pristine analogs [39]. In biological media, particles rapidly acquire a protein corona, modifying their surface charge and hydrodynamic diameter, which alters their uptake and apparent toxicity [40].

Another major bias arises from chemical impurities. Leachable additives in case of TWPs (e.g., plasticizers, antioxidants, metals) or adsorbed environmental contaminants can drive cytotoxicity independently of the polymer itself [12,60]. MPs and NPs can also act as Trojan Horses, transporting co-pollutants such as PAHs, antibiotics, endocrine disruptors or heavy metals into cells, thereby exaggerating apparent toxicity [7,9,61,62]. In addition, endotoxin contamination (e.g., bacterial LPS) can activate NF-κB-mediated inflammatory pathways and oxidative stress, producing false-positive toxicity results if not properly controlled by endotoxin-free protocols or reporter assays [40].

Experimental design further affects toxicological reliability. Many studies apply acute, unrealistically high doses (tens to hundreds of µg/mL), far exceeding environmentally relevant concentrations (ERCs) [14,23,54]. Such overexposure can trigger non-physiological responses and mask subtle, chronic effects. Likewise, submerged 2D cultures fail to reproduce the deposition and diffusion dynamics of airborne particles, leading to uneven exposure [54]. Advanced systems such as the air–liquid interface (ALI) and human airway organoids (hAOs) provide more realistic exposure scenarios and should be prioritized for assessing low-dose, chronic responses [38,40,54]. The absence of appropriate control particles (e.g., inert beads) further limits interpretation by confounding polymer-specific and shape-dependent effects [53].

In summary, toxicological results on MPs, NPs, and TWPs are affected by particle variability, chemical contamination, endotoxin interference, and unrealistic exposure conditions. To improve reproducibility and environmental relevance, studies must adopt standardized particle characterization, endotoxin testing, environmentally relevant doses, and physiologically realistic exposure models such as ALI and organoids.

### 4.4. Future Research Agenda

The growing body of evidence on the occurrence and toxicity of airborne MPs underscores the urgent need to establish standardized, reproducible, and environmentally relevant methodologies [5,14,28]. To ensure comparability and robustness of results, future studies should address the following research priorities listed below.

#### 4.4.1. Standardization of Exposure Metrics and Interlaboratory Comparability

Current MPs research suffers from the lack of harmonized sampling, analytical, and reporting protocols, which hampers direct comparison between laboratories [63]. Standardized workflows should be developed for all analytical stages-from sampling and extraction to identification and quantification-accompanied by rigorous quality assurance and control (QA/QC) procedures, including blanks and reference materials [5,28,64]. Reporting should consistently include both particle count and mass-based quantification, alongside detection limits (LOD) and quantification limits (LOQ) of analytical methods. Environmental metadata (sampling height, meteorological conditions, site type) must also be included to improve comparability and model calibration across studies [5].

#### 4.4.2. Incorporation of Aged and Weathered Particles

Most toxicological and adsorption studies still rely on pristine laboratory-grade MPs, which fail to represent environmentally aged materials [61]. Weathering processes (UV radiation, oxidation, hydrolysis, and mechanical abrasion) alter surface chemistry, polarity, and crystallinity, increasing roughness and reactive surface area [61]. Such transformations enhance pollutant adsorption and often amplify toxicity [3,7,61,62,65]. Future studies should therefore systematically examine aged MPs to better reflect real environmental exposure scenarios and understand their altered toxicodynamic behavior.

#### 4.4.3. Integration of “Omics”-Based Endpoints

Traditional toxicity assays (e.g., viability, ROS, cytokines) provide limited insight into molecular mechanisms [64]. Advanced multi-omics approaches—including toxicogenomics, proteomics, and metabolomics-should be applied to reveal pathways involved in oxidative stress, inflammation, apoptosis, and ferroptosis [64,66]. Metabolomics studies have already demonstrated metabolic reprogramming and mitochondrial dysfunction in liver and lung cells exposed to nanoplastics [13]. Tiered testing strategies combining omics data with conventional endpoints would improve mechanistic understanding and facilitate predictive toxicology [64].

#### 4.4.4. Environmentally Realistic Exposure Doses and Modeling

Most current in vitro and in vivo experiments apply concentrations several orders of magnitude higher than those found in real environments [67]. Future research should focus on environmentally relevant concentrations (ERCs), enabling the determination of no-observed-effect concentrations (NOECs) and realistic dose–response thresholds [67]. For inhalation exposure, emphasis should be placed on respirable fractions (PM_2.5_, PM_1_) and high-risk environments such as indoor air. Advanced atmospheric transport models (e.g., Lagrangian approaches) are also needed to link ambient levels with internal exposure doses [6]. Bridging the gap between experimental and real-world exposure will allow more accurate risk assessments and improve regulatory relevance [6].

In summary, future research should prioritize methodological standardization, incorporation of environmentally aged particles, adoption of omics endpoints, and the use of environmentally realistic exposure doses. Addressing these challenges is essential for developing a unified framework to assess human health risks associated with airborne MPs.

## 5. Conclusions

Microplastics (MPs) in airborne particulate matter (PM) represent a significant and emerging environmental and public health concern. As demonstrated in this review, MPs-particularly those derived from tire wear particles (TWPs), which dominate urban air-originate largely from road transport and other anthropogenic activities. Their ability to adsorb and transport hazardous substances, including metals and organic pollutants, further increases their toxic potential and persistence in the atmosphere. The analysis of MPs within PM is methodologically demanding, requiring multi-stage workflows that integrate representative sampling, efficient separation from the bulk matrix, and both qualitative and quantitative characterization. Commonly applied techniques such as filtration, density separation, enzymatic digestion, chemical oxidation, and electrostatic separation offer varying levels of efficiency depending on polymer type and PM composition. Despite recent analytical advances, the lack of standardized protocols continues to hinder reproducibility and interlaboratory comparability, thereby limiting the robustness of environmental assessments and exposure estimations.

In vitro studies have consistently confirmed the toxic potential of MPs toward human respiratory cells, including the induction of oxidative stress, inflammation, apoptosis, and even carcinogenic-related signaling. These findings raise serious concerns about long-term exposure in urban populations, particularly among vulnerable groups such as children and the elderly. Given the increasing atmospheric presence of MPs and their documented biological activity, international standardization of sampling and analytical methodologies is urgently needed. Furthermore, integrating advanced biological models-such as air–liquid interface (ALI) systems and human airway organoids-with epidemiological data will be crucial for elucidating mechanistic pathways of toxicity and improving health risk assessment.

Future research should aim to refine non-destructive separation and identification techniques, evaluate chronic health impacts using physiologically relevant human models, and address existing regulatory and methodological gaps related to airborne microplastic pollution. Expanding biomonitoring programs and applying natural bioindicators may further enhance exposure assessment. Overall, this review emphasizes that tackling the issue of airborne MPs requires a multidisciplinary, collaborative, and globally coordinated approach, bringing together environmental scientists, toxicologists, policymakers, and public health professionals to establish unified strategies for monitoring, risk evaluation, and mitigation.

## Figures and Tables

**Table 1:** Summary of sampling, digestion, and density separation methods for airborne microplastics (MPs), with focus on reagents, analytical applicability, and QA/QC limitations.

Method	Reagent/Filter	Description	Advantages/Application	Limitations Reported	Ref.
Sample collection	Quartz fiber, glass fiber, PTFE filters	Filtration of airborne PM to collect MPs on solid substrate. Filters pre-cleaned and baked at 550 °C to remove organic residues.	Commonly used for PM_10_, PM_2.5_ samplers; compatible with microscopic and spectroscopic analysis.	Risk of airborne contamination during sampling and handling; fibers from clothing may interfere; procedural blanks essential.	[5]
Digestion	H_2_O_2_	Mild oxidation of organic matter; reaction accelerated by heating (50–60 °C).	Effective for removing natural organics with minimal polymer degradation; environmentally safe.	Incomplete digestion for lipid-rich samples; may slightly deform MPs at high temp.	[34]
	H_2_O_2_ + Fe^2+^	Radical-based oxidation; strong removal of biological residues.	Highly effective for organic matrices; short reaction time.	May oxidize polymer surfaces and alter FTIR/Raman spectra; reaction exothermic—temp control required.	[5,34]
	KOH	Hydrolytic removal of proteins and fats; long incubation (24–48 h at 40 °C).	Suitable for organic-rich samples (e.g., indoor dust).	May damage polyamide and polyester; prolonged contact affects morphology.	[34]
	NaOH	Similar to KOH but less effective for lipids.	Inexpensive, widely available.	Degrades PET and PVC; unsuitable for long digestion periods.	[34]
	Enzymatic	Enzymatic hydrolysis of cellulose, fats, and proteins.	Gentle, preserves polymer integrity and color; ideal for QA/QC validation.	Time-consuming and costly; enzyme activity depends on pH/temp.	[34]
	Acidic	Strong acid digestion to remove mineral/organic matter.	Rapid cleaning of heavily contaminated samples.	Causes polymer degradation and color loss; unsuitable for spectroscopy.	[28]
Density separation	NaCl	MPs float in saline while heavier particles settle.	Cheap, safe, easy disposal; good for PE/PP.	Inefficient for dense polymers (PET, PVC); may underestimate MPs.	[35]
	CaCl_2_	Medium-density separation for mixed MP types.	Simple preparation; reusable solution.	Crystallization on filters affects weighing; incomplete separation for very dense MPs.	[35]
	NaI	High-density solution ensures recovery of heavy polymers.	Effective for PET, PVC, and composite MPs.	Expensive, toxic; must be filtered/recycled; may contaminate FTIR background.	[34,35]
	ZnCl_2_	Very dense liquid allowing full recovery of all polymers.	High efficiency; suitable for complex PM matrices.	Highly corrosive and environmentally hazardous; disposal issues.	[34]
QA/QC controls	Reference materials/ clean filter	Control samples analyzed in parallel to environmental samples.	Detects and corrects background contamination.	Neglecting blanks leads to false positives; synthetic clothing should be avoided.	[28,34]

**Table 2:** Summary of previous studies investigating the toxicological effects of microplastics (MPs).

Endpoints	Key Findings	Limitations Reported	Ref.
Cell viability, ROS production, cytokine secretion, barrier integrity.	Dose-dependent cytotoxicity and oxidative stress; disruption of epithelial barrier; increased pro-inflammatory signaling.	Pristine MPs used; exposure concentrations exceed environmental levels; short exposure duration.	[37]
ROS generation, mitochondrial function, EMT markers.	Surface chemistry modulates toxicity; amine-modified particles induce stronger ROS and EMT-related changes.	Surface chemistry is not representative of environmental MPs; limited exposure durations; no chronic studies.	[38]
Cell viability, oxidative stress, inflammatory cytokine expression, apoptosis.	Consistent oxidative stress and inflammation across studies; apoptosis responses vary by polymer type.	Focus on A549 cells limits extrapolation; large variability in study designs.	[39]
Cytokine secretion, oxidative damage, DNA strand breaks.	Polystyrene nanoparticles trigger inflammatory responses and genotoxicity in bronchial epithelial cells.	Nanoplastics studied do not reflect environmental weathering; artificial culture conditions.	[40]
ROS production, apoptosis, mitochondrial damage.	Environmental MPs induce higher oxidative damage compared to pristine particles; weathering increases toxicity.	Limited particle characterization; lack of dose–response curve for environmentally derived MPs.	[41]
Inflammatory cytokine expression, oxidative stress biomarkers.	Airborne PM-bound MPs induce stronger inflammatory signaling than isolated MPs; synergistic pollutant effects observed.	PM matrix complexity makes causal attribution difficult; short-term exposures only.	[42]
Cell viability, oxidative stress, autophagy markers.	MPs disrupt autophagy processes, contributing to oxidative stress and cell death.	Mechanistic link between autophagy disruption and in vivo effects remain speculative.	[43]
Oxidative stress, inflammatory mediator release, epithelial barrier function.	Barrier integrity compromised; increased IL-8 and TNF-α production; oxidative stress dose-dependent.	Only pristine MPs tested; relevance to inhaled environmental MPs uncertain.	[44]
Pulmonary toxicity mechanisms (ROS, inflammation, fibrosis).	Summarizes mechanistic pathways from in vitro and in vivo evidence; highlights chronic exposure risks.	Mechanistic evidence largely from high-dose exposures; nanoplastics poorly represented.	[45]
Respiratory health outcomes in in vivo and in vitro models.	Evidence supports pulmonary toxicity from inhaled MPs; outcomes vary by particle size, polymer type, and exposure route.	Heterogeneity in experimental conditions; environmental exposure levels underrepresented.	[46]

**Table 3:** Comparative summary of selected experimental studies on the cytotoxicity, oxidative stress, and mechanistic responses induced by MPs, NPs and tire wear particles (TWPs) in various biological models.

Polymer/ Type/Size	Dose	Model	Key Endpoints	Key Limitations	Ref.
PS-NPs (unmodified, –NH_2_, –COOH), 80 nm	2.5–400 µg·mL^−1^	A549 (submerged)	Decreased cell viability; induction of micronuclei (genotoxicity); increased reactive oxygen species (oxidative stress)	High doses; pristine spherical NPs; lack of aging; short exposure; limited mechanistic data	[39]
PS-NPs 25 nm and 70 nm	Concentration-dependent (dose N/A)	A549 (submerged)	Enhanced cellular uptake for 25 nm particles; induction of apoptosis; S-phase cell-cycle arrest; upregulation of IL-8 and TNF-α	Simplified model; pristine PS; short-term exposure	[45]
PS-MPs 0.5 and 5 µm	10 mg·L^−1^ (in drinking water)	Mouse C57BL/6J (in vivo, 3-month)	Reduction in body-to-liver weight ratio; increased oxidative stress; decreased antioxidant enzyme activity; ultrastructural hepatic damage (stronger for 5 µm particles)	Not inhalation model; in vivo only; mechanism (SIRT3/SOD2) partly identified	[58]
PS (9.5–11.5 µm), PE (1–4 µm)	1, 10, 20 µg·mL^−1^	MDCK; L929 (submerged)	Dose-dependent reduction in viability; increased metabolic activity; upregulation of SOD3 and TNF-α; downregulation of IL-1β	Non-pulmonary lines; pristine microspheres; mechanistic data limited	[50]
PS MPs/NPs (25–100 nm range)	Variable (µg·mL^−1^)	A549; BEAS-2B (submerged)	Enhanced ROS generation; mitochondrial dysfunction; DNA strand breaks; size-dependent cellular internalization	High acute doses; pristine particles	[59]
Tire Wear Particles (TWPs) ≈ 100 nm	Up to 200 µg·mL^−1^	Human airway organoids (hAOs from HBECs)	Inhibition of organoid growth; increased apoptosis and ROS generation (2.8-fold); upregulation of IL-6, TNF-α, CAT, and SOD2; downregulation of KRT5 and SCGB1A1	Complex mixture (additives, metals); unclear dose–exposure translation; short exposure time	[27]
PS-NPs 156 ± 12 nm	1–1000 ng·cm^−2^	BEAS-2B (Transwell, quasi-ALI)	Cytotoxic effects at ≥10 ng·cm^−2^; oxidative stress at ≥1 ng·cm^−2^; activation of NF-κB, NLRP3, and ROCK-1; induction of apoptosis and autophagy at higher doses	Pristine PS; short-term; needs chronic low-dose validation; limited dose-response data	[41]
PS-NPs 800 nm	10–500 µg·mL^−1^	A549 (submerged)	Decreased viability; increased H_2_O_2_ production; upregulation of senescence markers (CDKN1A, IL1A/B, IL6, CXCL8); activation of apoptotic genes (BAX, CASP3, BCL2)	High doses; submerged culture; short exposure duration	[44]

## Data Availability

No new data were created or analyzed in this study. Data sharing is not applicable to this article.
